# A matter of sex—persistent predictive value of MECKI score prognostic power in men and women with heart failure and reduced ejection fraction: a multicenter study

**DOI:** 10.3389/fcvm.2024.1390544

**Published:** 2024-07-03

**Authors:** Giulia Grilli, Elisabetta Salvioni, Federica Moscucci, Alice Bonomi, Gianfranco Sinagra, Michele Schaeffer, Jeness Campodonico, Massimo Mapelli, Maddalena Rossi, Cosimo Carriere, Michele Emdin, Massimo Piepoli, Stefania Paolillo, Michele Senni, Claudio Passino, Anna Apostolo, Federica Re, Caterina Santolamazza, Damiano Magrì, Carlo M. Lombardi, Ugo Corrà, Rosa Raimondo, Antonio Cittadini, Annamaria Iorio, Andrea Salzano, Rocco Lagioia, Carlo Vignati, Roberto Badagliacca, Andrea Passantino, Pasquale Perrone Filardi, Michele Correale, Enrico Perna, Davide Girola, Marco Metra, Gaia Cattadori, Marco Guazzi, Giuseppe Limongelli, Gianfranco Parati, Fabiana De Martino, Maria Vittoria Matassini, Francesco Bandera, Maurizio Bussotti, Angela Beatrice Scardovi, Susanna Sciomer, Piergiuseppe Agostoni, MECKI Score Research Group, Armando Ferraretti

**Affiliations:** ^1^ Heart Failure Unit Centro Cardiologico Monzino, IRCCs, Milan, Italy; ^2^ Department of Clinical Medicine and Medical Specialties, Azienda Ospedaliera Universitaria Policlinico Umberto I, Rome, Italy; ^3^ Cardiothoracovascular Department, Azienda Sanitaria Universitaria Giuliano Isontina (ASUGI) and University of Trieste, Trieste, Italy; ^4^ Research Group for Rehabilitation in Internal Disorders, Department of Rehabilitation Sciences, KU Leuven, Leuven, Belgium; ^5^ Department of Clinical Sciences and Community Health, Cardiovascular Section, University of Milan, Milan, Italy; ^6^ Health Science Interdisciplinary Center, Scuola Superiore Sant’Anna, Pisa, Italy; ^7^ Cardio-Thoracic Department, Fondazione Toscana Gabriele Monasterio, Pisa, Italy; ^8^ Clinical Cardiology, IRCCS Policlinico San Donato, Milan, Italy; ^9^ Department of Biomedical Sciences for Health, University of Milan, Milan, Italy; ^10^ Dipartimento di Scienze Biomediche Avanzate, Federico II University of Naples, Naples, Italy; ^11^ Cardiovascular Department, Cardiology Unit, ASST Papa Giovanni XXIII, Bergamo, Italy; ^12^ Cardiology Division, Cardiac Arrhythmia Center and Cardiomyopathies Unit, San Camillo-Forlanini Hospital, Roma, Italy; ^13^ Dipartimento Cardio-Toraco-Vascolare, Ospedale Cà Granda-A.O. Niguarda, Milano, Italy; ^14^ Department of Clinical and Molecular Medicine, Azienda Ospedaliera Sant’Andrea, “Sapienza” Università Degli Studi di Roma, Roma, Italy; ^15^ Cardiology, Department of Medical and Surgical Specialities, Radiological Sciences, and Public Health, University of Brescia, Brescia, Italy; ^16^ Cardiology Department, Istituti Clinici Scientifici Maugeri, IRCCS, Veruno Institute, Veruno, Italy; ^17^ U.O. Prevenzione e Riabilitazione Cardiovascolare, IRCCS, Ospedale San Raffaele, Milano, Italy; ^18^ Department of Translational Medical Sciences, Federico II University, Naples, Italy; ^19^ Interdepartmental center for gender medicine research ‘GENESIS’, Federico II University, Naples, Italy; ^20^ Cardiac Unit, AORN A Cardarelli, Naples, Italy; ^21^ Department of Cardiovascular Sciences, University of Leicester, Leicester, United Kingdom; ^22^ UOC Cardiologia di Riabilitativa, Mater Dei Hospital, Bari, Italy; ^23^ Dipartimento di Scienze Cliniche, Internistiche, Anestesiologiche e Cardiovascolari, “Sapienza”, Rome University, Rome, Italy; ^24^ Division of Cardiology, Istituti Clinici Scientifici Maugeri, Institute of Bari, Bari, Italy; ^25^ Department of Advanced Biomedical Sciences, Federico II University of Naples and Mediterranea CardioCentro, Naples, Italy; ^26^ Department of Cardiology, University of Foggia, Foggia, Italy; ^27^ Clinica Hildebrand, Centro di Riabilitazione Brissago, Brissago, Switzerland; ^28^ Department of Medical and Surgical Specialities, Radiological Sciences and Public Health, University of Brescia Medical School, Brescia, Italy; ^29^ Unità Operativa Cardiologia Riabilitativa, IRCCS Multimedica, Milano, Italy; ^30^ Department of Cardiology, University of Milano School of Medicine, San Paolo Hospital, Milano, Italy; ^31^ Cardiologia SUN, Ospedale Monaldi (Azienda dei Colli), Seconda Università di Napoli, Napoli, Italy; ^32^ Department of Cardiovascular, Neural and Metabolic Sciences, San Luca Hospital, Istituto Auxologico Italiano, IRCCS, Milan, Italy; ^33^ Department of Medicine and Surgery, University of Milano-Bicocca, Milan, Italy; ^34^ Unità Funzionale di Cardiologia, Casa di Cura Tortorella, Salerno, Italy; ^35^ Cardiac Intensive Care Unit-Cardiology Division, Cardiovascular Department, Ospedali Riuniti di Ancona, Ancona, Italy; ^36^ Department of Biomedical Sciences for Health, University of Milano, Milan, Italy; ^37^ Cardiology University Department, IRCCS Policlinico San Donato, Milan, Italy; ^38^ Cardiac Rehabilitation Unit, Istituti Clinici Scientifici Maugeri, IRCCS, Scientific Institute of Milan, Milan, Italy; ^39^ Cardiology Division, Santo Spirito Hospital, Roma, Italy

**Keywords:** heart failure with reduced ejection fraction, prognosis, sex, MECKI score, risk

## Abstract

**Background:**

A sex-based evaluation of prognosis in heart failure (HF) is lacking.

**Methods and results:**

We analyzed the Metabolic Exercise test data combined with Cardiac and Kidney Indexes (MECKI) score registry, which includes HF with reduced ejection fraction (HFrEF) patients. A cross-validation procedure was performed to estimate weights separately for men and women of all MECKI score parameters: left ventricular ejection fraction (LVEF), hemoglobin, kidney function assessed by Modification of Diet in Renal Disease, blood sodium level, ventilation vs. carbon dioxide production slope, and peak oxygen consumption (peakVO_2_). The primary outcomes were the composite of all-cause mortality, urgent heart transplant, and implant of a left ventricle assist device. The difference in predictive ability between the native and sex recalibrated MECKI (S-MECKI) was calculated using a receiver operating characteristic (ROC) curve at 2 years and a calibration plot. We retrospectively analyzed 7,900 HFrEF patients included in the MECKI score registry (mean age 61 ± 13 years, 6,456 men/1,444 women, mean LVEF 33% ± 10%, mean peakVO_2_ 56.2% ± 17.6% of predicted) with a median follow-up of 4.05 years (range 1.72–7.47). Our results revealed an unadjusted risk of events that was doubled in men compared to women (9.7 vs. 4.1) and a significant difference in weight between the sexes of most of the parameters included in the MECKI score. S-MECKI showed improved risk classification and accuracy (area under the ROC curve: 0.7893 vs. 0.7799, *p* = 0.02) due to prognostication improvement in the high-risk settings in both sexes (MECKI score >10 in men and >5 in women).

**Conclusions:**

S-MECKI, i.e., the recalibrated MECKI according to sex-specific differences, constitutes a further step in the prognostic assessment of patients with severe HFrEF.

## 1 Introduction

Heart failure (HF) represents a global epidemic with a poor prognosis and a 5-year mortality up to 50% despite significant advances in pharmacological, device, and surgical interventions. At present, approximately 64.3 million people are estimated to live with HF worldwide and women account for up to half of the prevalent cases (1). According to the high prevalence of HF in the female population, a sex difference in clinical management is ascertained and addressed by the most recent American and European guidelines (2, 3). In particular, women are less likely to receive optimal medical therapy or be referred to specialty care and are less likely to receive device therapy or heart transplantation (HT) (4–6). It has also been shown that women with HF have a lower quality of life than men, with more functional capacity impairment, prolonged hospital stay, and depression, but an overall better survival (7). The cause for sex differences in morbidity and mortality remains mostly unknown; however, it can be partially addressed by the different etiologies and phenotypes.

As shown in community-based cohort studies, women are more likely than men to have HF with preserved left ventricular (LV) systolic function and less frequently to have ischemic cardiomyopathy (8). In the Swede HF registry (4), women account for 55% of all cases of HF with preserved ejection fraction (HFpEF) and only 29% of all cases of HF with reduced ejection fraction (HFrEF). The higher percentage of women with HFpEF in observational studies may partly be the result of the age distribution of the population at risk, as they have a higher life expectancy (4, 8) and tend to develop HF at an older age compared to men (9). The older age at the time of diagnosis comes with a higher burden of comorbidities, family and childcare responsibilities, and financial, cultural, and socioeconomic barriers. These age-related characteristics in the context of the low prevalence of women in the HFrEF population can partially explain the under-representation of women in clinical trials and prediction score models, raising concerns regarding the generalizability of both trial results and prognostic models (10). Of note, the female sex still represents a significant predictor of improved survival in patients with HFrEF (11, 12) despite a comparatively low peak oxygen consumption (peakVO_2_) at cardiopulmonary exercise testing (CPET).

Validated prognostic risk models represent a valuable tool to quantify survival prospects to patients and care providers and may help in decision making and directing care in HF (7, 13). Unfortunately, the current prognostic scores in HF lack a true specific sex-oriented assessment behind a generic adjustment, consisting of a few variables used to assess the severity of HF, such as data derived from CPET and kidney function. In this regard, in 2019, Vishram-Nielsen et al. set out to examine the predictive performance of the SHFM and MAGGIC scores separately in men and women revealing an overall similar discriminatory capacity with similar predicted vs. observed risk between sexes. This held for mortality and for a composite endpoint of mortality, implantation of a ventricular assist device, and/or transplantation. However, both scores overestimated mortality at 3 years in women (14). Nevertheless, when applying risk prediction models, it is always important to take into consideration sex differences in predictive risk factors and outcomes (15). In 2013, the Metabolic Exercise test data combined with Cardiac and Kidney Indexes (MECKI) score was proposed by an Italian working group, to identify the risk of cardiovascular mortality and urgent heart transplantation (16, 17). It relies on six variables: hemoglobin (Hb), sodium (Na^+^), kidney function by means of the Modification of Diet in Renal Disease (MDRD) equation, left ventricular ejection fraction (LVEF) by echocardiography, percentage of predicted peakVO_2_, and minute ventilation-carbon dioxide production (VE/VCO_2_) slope. The MECKI score has been validated in patients affected by HFrEF and it showed a high accuracy in the absolute risk prediction of cardiac events, with very high area under the receiver operating characteristic (ROC) curve (AUC) (17). Previous studies on the MECKI score database have demonstrated that female survival advantage is lost when sex-specific differences are correctly considered (18); however, sex-specific differences in MECKI score prognostic power as well as differences in the weight of the single parameters included in the MECKI score are unknown (19).

## 2 Methods

The present study analyzed the MECKI score registry population enrolled between 1993 and 2022 across 26 Italian sites (17). In brief, the inclusion criteria for the MECKI study were previous or present symptoms of HF and history or presence of LVEF < 40%, unchanged HF medications for at least 3 months, ability to perform a CPET, and no major cardiovascular treatment or intervention scheduled (17). For inclusion in the registry, it is mandatory that the patients performed a CPET using the following modalities: (1) exercise with a progressively increasing workload on an electronically braked cycle ergometer or a treadmill with a protocol set to reach peak exercise in 8–12smin (20); and (2) symptom-limited tests. Ventilation and respiratory gases were collected breath by breath and analyzed following a standard technique (21). Similarly, peakVO_2_ and ventilation vs. CO_2_ production slope (VE/VCO_2_ slope) were calculated as standard (21). The percentage of predicted value (peakVO_2_%) is reported according to Hansen et al. (22). In addition to CPET-related variables, the MECKI registry collects echocardiographic (ECG), pharmacological therapy, and blood chemistry data at enrollment, as well as vital status and causes of events during follow-up. Patient follow-up and data management procedures were performed as previously described (17). For prognostic evaluation, the end point was the composite of cardiovascular death, urgent HT, or implantation of a left ventricle assist device (LVAD). The study was approved by Centro Cardiologico Monzino-IEO ethical committee (protocol number: CCM04_21 PA).

### 2.1 Statistical analysis

All continuous variables have a normal distribution, and these variables are presented as means and standard deviation. Categorical data are reported as frequencies and percentages. Group comparisons for continuous and categorical variables were performed using *t*-tests and chi-square (χ^2^) tests, respectively. To obtain consistent betas as a weight of each individual variable (Beta) included in the score, a cross-validation procedure was used. Specifically, the sample of men and women separately was randomly divided in half 200 times: in the first half (training set) the betas for each variable were estimated by implementing a logistic model and then tested on the second half (testing set) by calculating the AUC. The mean of the 200 betas obtained from the 200 cross-validation procedures was used as the weight for each variable in the MECKI score, separately by sex. Sex differences in the weights of each variable were identified by comparing the 95% confidence intervals of the betas. Standardized betas in men and women were also calculated.

Calibration of the MECKI algorithm was evaluated by dividing the sample into deciles of risk and by comparing the observed events with the predicted events in each decile (Hosmer–Lemeshow test). The comparison between the sex recalibrated MECKI (S-MECKI) score and native MECKI score was carried out by calculating the AUC and comparing using the De Long test. A *p*-value <0.05 was considered statistically significant.

## 3 Results

We considered a total of 7,900 patients with HFrEF. Table 1 reports the characteristics of the population according to sex. Specifically, female HF patients had less frequent ischemic origin of HF and showed higher LVEF, lower peakVO_2_ but higher VO_2_% pred, and lower Hb concentration. Regarding treatment, female HF patients were less frequently implanted with cardioverter-defibrillator (ICD) or cardiac resynchronization therapy (CRT), and less aggressively treated with angiotensin-converting enzyme inhibitors (ACEi), angiotensin II type 1 receptor blockers (AT1b), angiotensin receptor-neprilysin inhibitors (ARNI), diuretics, and, but only as a trend, less frequently received β-blockers and mineralocorticoid receptor antagonists (MRA).

**Table 1:** Study population characteristics.

	Females	Males	*p*
	*n* = 1,444	*n* = 6,456	
Age (years)	61.7 ± 13.7	61.6 ± 12.4	0.713
BMI (kg/m^2^)	25.7 ± 4.9	26.9 ± 4.2	<0.001
LVEF (%)	36.4 ± 11.4	32.3 ± 10.0	<0.001
SBP (mmHg)	116.8 ± 17.8	116.6 ± 17.4	0.809
Rest HR (bpm)	71.6 ± 12.8	70.2 ± 12.5	<0.001
PeakVO_2_ (ml/min)	881 ± 296	1,214 ± 440	<0.001
PeakVO_2_ (ml/min/kg)	13.3 ± 4.3	15.0 ± 5.0	<0.001
PeakVO_2_ (% of predicted)	63.2 ± 18.3	54.6 ± 17.0	<0.001
Peak HR (bpm)	119 ± 26	118 ± 25	0.025
VE/VCO_2_slope	33.5 ± 7.7	33.2 ± 7.9	0.153
MDRD (ml/min/1.73 m^2^)	68.6 ± 24.6	72.3 ± 24.2	<0.001
Hemoglobin (g/dl)	12.7 ± 1.4	13.7 ± 1.7	<0.001
Na^+^ (mmol/L)	139.6 ± 3.0	139.4 ± 3.3	0.067
NYHA class (*n*, %)			
NYHA 1	178 (12.5%)	1,035 (16.1%)	0.005
NYHA 2	831 (58.1%)	3,585 (55.9%)	
NYHA 3	405 (28.3%)	1,710 (26.6%)	
NYHA 4	16 (1.1%)	88 (1.4%)	
Atrial fibrillation (*n*, %)	227 (15.7%)	1,158 (18%)	0.042
ICD (*n*, %)	356 (24.7%)	2,291 (35.5%)	<0.001
CRT (n, %)	165 (11.5%)	957 (15%)	0.001
Etiology (*n*, %)			
Idiopathic	641 (48.4%)	2,409 (40.0%)	<0.001
Ischemic	330 (24.9%)	2,988 (49.7%)	
Valvular	116 (8.8%)	217 (3.6%)	
Other	238 (18%)	404 (7%)	
ACEi/AT1b/ARNI (*n*, %)	1,289 (89.3%)	5,958 (92.3%)	<0.001
Diuretics (*n*, %)	1,107 (77.9%)	5,088 (80.4%)	0.036
Statin (*n*, %)	522 (37.4%)	3,039 (48.8%)	<0.001
Allopurinol (*n*, %)	278 (19.9%)	1,837 (29.1%)	<0.001
MRA (*n*, %)	702 (50.3%)	3,353 (53.1%)	0.053
Antiplatelets (*n*, %)	652 (46.5%)	3,494 (55.1%)	<0.001
Oral anticoagulant (*n*, %)	385 (27.4%)	2,087 (32.9%)	<0.001
Digoxin (*n*, %)	220 (16.2%)	1,194 (19.8%)	0.003
Amiodarone (*n*, %)	256 (18.9%)	1,580 (26.1%)	<0.001
Beta-blockers (*n*, %)	1,230 (86.0%)	5,628 (87.9%)	0.057

BMI, body mass index; SBP, systolic blood pressure; HR, heart rate; VE/VCO_2_ slope, ventilation vs. metabolic production of carbon dioxide relationship slope; Na^+^, sodium; NYHA, New York Heart Association class.

Table 2 shows the average betas of the six parameters that generate the original MECKI score algorithm: LVEF, Hb, kidney function assessed by MDRD, blood Na^+^ level, VE/VCO_2_ slope, and peakVO_2_ obtained by the cross-validation procedure. A similar beta value between men and women was observed for MDRD and VE/VCO_2_ slope while a statistically significant higher weight was observed for LVEF, Na^+^, and peakVO_2_ in men and for Hb in women. The unadjusted risk of an event (cardiovascular death, urgent heart transplant, or LVAD) was doubled in men compared to women.

**Table 2:** Cross-validation of each MECKI score variable on prognosis (means of 200 repetitions used to estimate the weight).

	Females			Males	
	Mean	95% IC		Mean	95% IC
Intercept	4.111	3.116 to 5.107	<	9.79	9.465 to 10.115
LVEF	−0.027	−0.025 to −0.03	<	−0.036	−0.035 to −0.037
Hb	−0.172	−0.158 to −0.186	>	−0.078	−0.073 to −0.084
MDRD	−0.009	−0.007 to −0.01	=	−0.011	−0.01 to −0.011
Na^+^	−0.017	−0.01 to −0.024	<	−0.059	−0.057 to −0.061
VE/VCO_2_ slope	0.025	0.022 to 0.027	=	0.028	0.027 to 0.029
PeakVO_2_ (% of predicted)	−0.034	−0.032 to −0.035	<	−0.047	−0.046 to −0.047

VE/VCO_2_ slope, ventilation vs. metabolic production of carbon dioxide relationship slope; Na^+^, sodium.

These results were used to create an S-MECKI score, using separate weights for each sex, as follows:

$$S\text{-}\mathrm{MECKI}=e^{\mathrm{esp}}/(1+e^{\mathrm{esp}}),$$

where, if female:

$$\mathrm{esp}=4.1116831+-0.0341452\times \mathrm{peakV}O_{2}\text{\%}\;\mathrm{pred}+0.0252531\times \mathrm{VE}/\mathrm{VC}O_{2}\;\mathrm{slope}+-0.1724513\times \mathrm{Hb}+-0.0175751\times Na^{+}+-0.0279113\times \mathrm{LVEF}+-0.0090766\times \mathrm{MDRD};$$

and if male:

$$\mathrm{esp}=9.790738+-0.0472631\times \mathrm{peakV}O_{2}\text{\%}\;\mathrm{pred}+0.0285722\times \mathrm{VE}/\mathrm{VC}O_{2}\;\mathrm{slope}+-0.0789262\times \mathrm{Hb}+-0.0597067\times Na^{+}+-0.0368194\times \mathrm{LVEF}+-0.0110881\times \mathrm{MDRD}.$$

**Figure 1:**
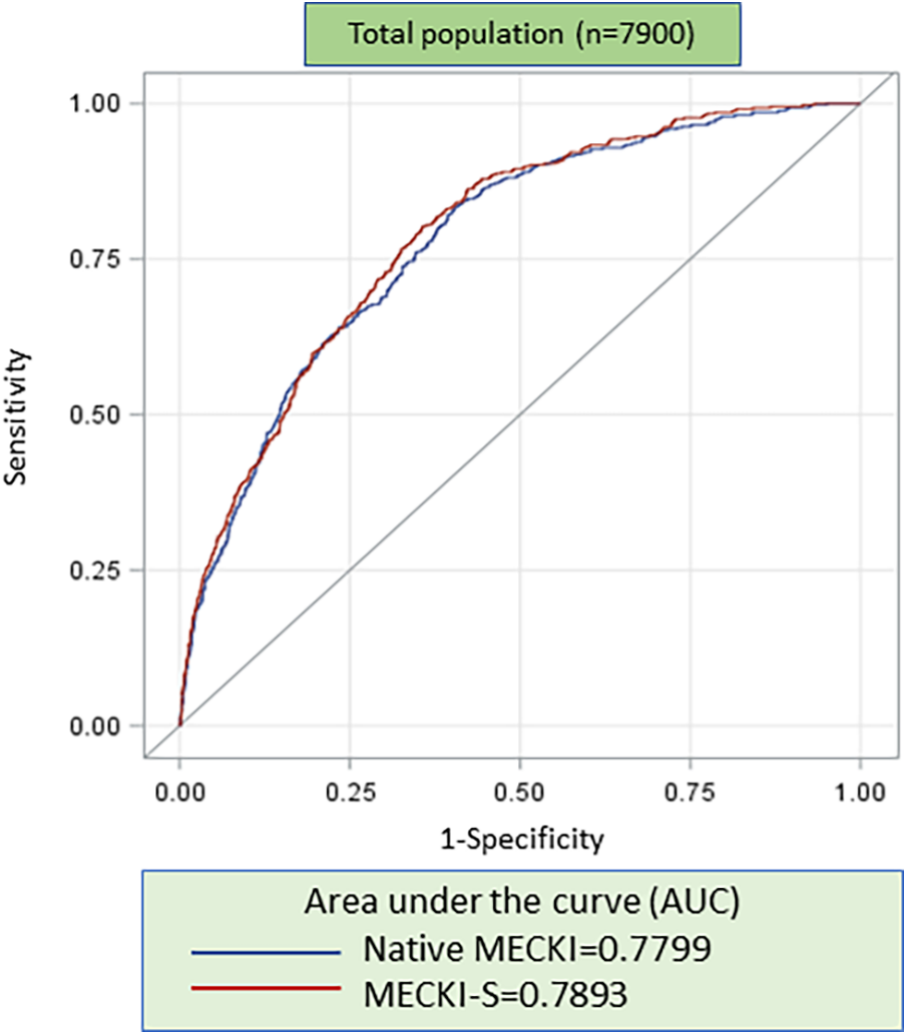
ROC analysis of native MECKI vs. S-MECKI scores in the whole study population.

**Figure 2:**
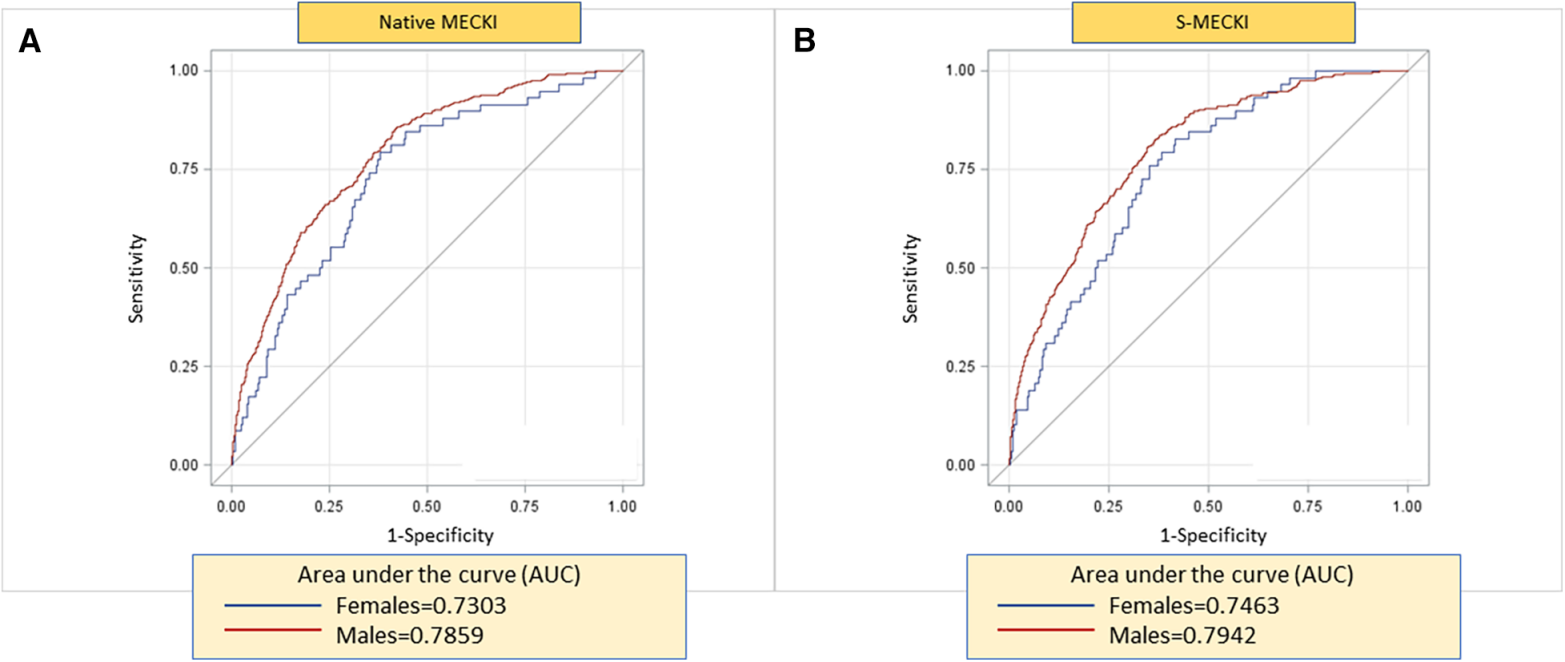
ROC analysis of native MECKI (**A**) vs. S-MECKI (**B**) scores.

In the overall population, the AUC of the S-MECKI score was slightly but significantly higher than the native MECKI score (*p* = 0.019), as shown in Figure 1. The AUC of the male and female populations analyzed separately for the native and S-MECKI scores are shown in Figure 2. The AUC of the MECKI score appears higher in the male sex using either the MECKI native formula or the sex-corrected formula. For both sexes, the MECKI sex-corrected formula showed a slight and similar prognostic improvement. The AUCs considering separately each variable included in the MECKI score are reported in Supplementary Figure 1. In both men and women, the highest AUC was for the MECKI score and among MECKI score variables for peakVO_2_, while the lowest was for Na^+^. All MECKI score variables standardized betas are reported in Table 3. In women, the highest weight was for peakVO_2_ followed by Hb, while in men it was peakVO_2_ followed by LVEF. The lowest weight was for Na^+^ and VE/VCO_2_ slope in women and Na^+^ and Hb in men.

**Table 3:** Standardized β for men and women.

	Standardized β (Females)	95% IC
PeakVO_2_ (% of predicted)	−0.591	−0.932 to −0.250
VE/VCO_2_ slope	0.193	−0.050 to 0.435
Hb	−0.312	−0.675 to 0.049
Na^+^	−0.076	−0.353 to 0.201
LVEF	−0.252	−0.517 to 0.0139
MDRD	−0.224	−0.519 to 0.071
	Standardized β (Males)	95% IC
PeakVO_2_ (% of predicted)	−0.819	−0.994 to −0.644
VE/VCO_2_ slope	0.226	0.122 to 0.329
Hb	−0.149	−0.279 to −0.019
Na^+^	−0.194	−0.293 to −0.096
LVEF	−0.384	−0.521 to −0.248
MDRD	−0.260	−0.379 to −0.1412

VE/VCO_2_ slope, ventilation vs. metabolic production of carbon dioxide relationship slope; Na^+^, sodium.

**Figure 3:**
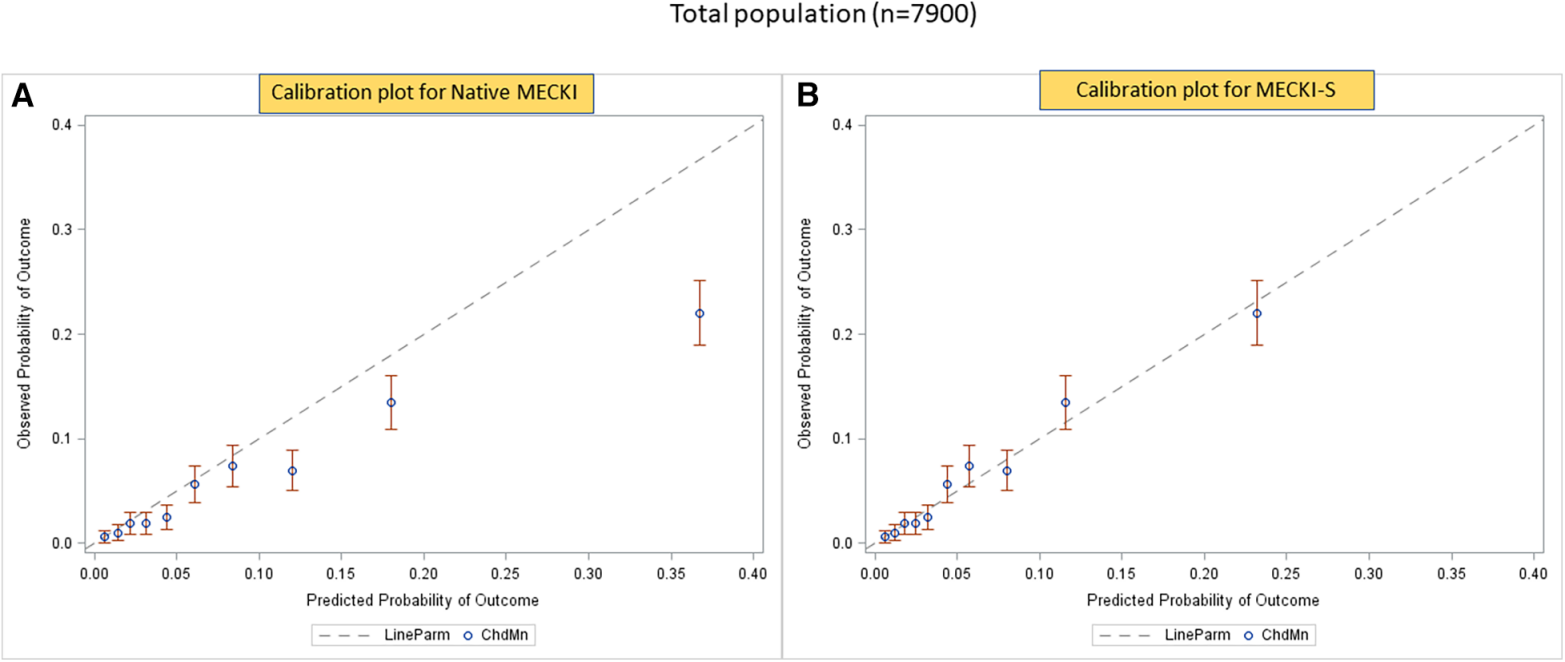
Calibration plots of native MECKI (**A**) and S-MECKI (**B**) scores in the whole study population.

**Figure 4:**
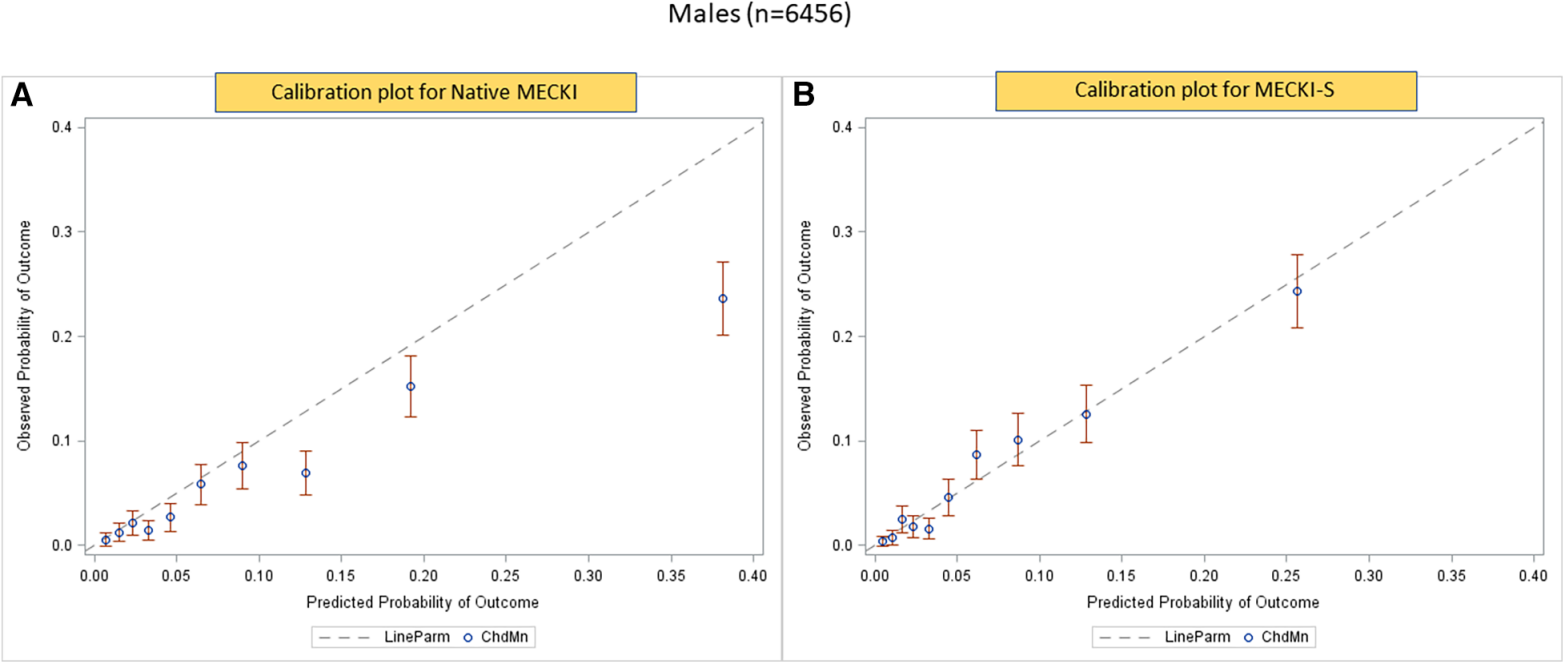
Calibration plots of native (**A**) and S-MECKI (**B**) scores in the male subgroup.

**Figure 5:**
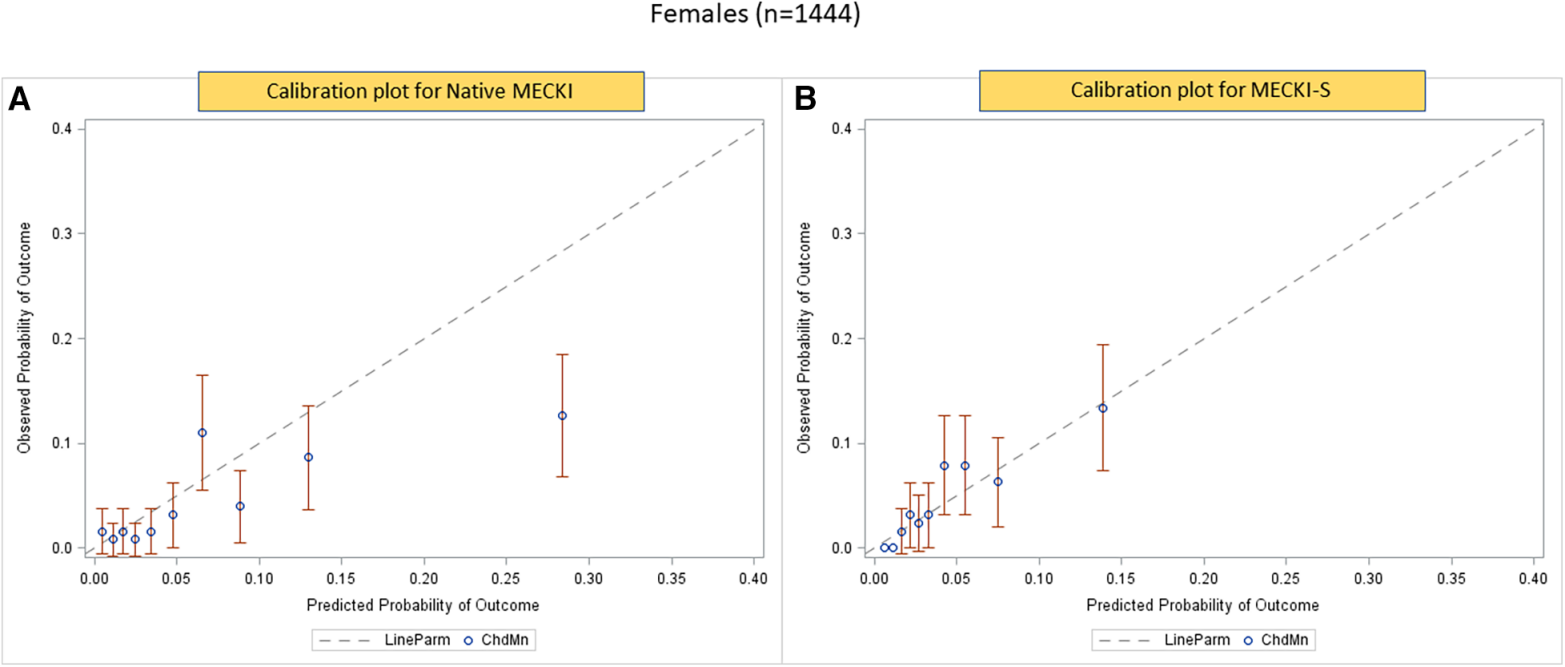
Calibration plots of native MECKI (**A**) and S-MECKI (**B**) scores in the female subgroup.

The calibration plots obtained using the native MECKI score (panel A) and the S-MECKI score (panel B) are shown in Figure 3, with the latter superior in the general population as well as sex-specific populations (Figures 4, 5). Notably, a greater prognostic improvement was observed in patients at higher risk of mortality or urgent transplant and specifically when MECKI score was >10% in men and >5% in women.

## 4 Discussion

In the present study, we showed that in the high-risk setting of HFrEF, the performance of the recalibrated MECKI score, the S-MECKI, was slightly superior to the native MECKI score due to better risk classification within 2 years, when the native MECKI score underestimates the risk of the composite endpoint of cardiovascular death, urgent HT, and implantation of a LVAD. This applies to the overall population as well as for a comparable entity to both men and women. Notably, this study has analyzed for the first time the specific contribution (weight) of each single variable of the score, revealing which are the parameters more influenced by sex.

Re-evaluation of prediction models is a desirable approach when evidence is found that raises doubts about the accuracy of a model in particular subgroups of patients or in specific conditions as for high-risk cases. Recalibration is considered an adequate approach to improve the accuracy of the prediction of the absolute risk (23). In the present study, we showed that recalibration of the MECKI score is advisable in high-risk patients, both in men and women, and particularly in elderly patients. This is likely related to the low number of cases with severe HF present in the original MECKI score data, which were used to build the MECKI score algorithm (17). Moreover, the AUC of the sex-calculated MECKI score, recalibrated according to the weighted variables, was significantly higher compared to the native MECKI score both when applied to the whole study population and separately to the sex-based subgroups (Figures 1, 2). Of note, CPET-derived parameters peakVO_2_ and VE/VCO_2_ slope showed the strongest prognostic power among the MECKI score variables (Supplementary Figure 1), confirming the pivotal role of exercise-derived parameters in the prognosis of HF.

The S-MECKI score also showed a greater accuracy in risk classification with an improved re-classification of patients in both the whole study population and in the sex-based subgroups, which was noticeable for patients at higher risk of events (Figures 3–5), specifically when the original MECKI score was >10 in men and >5 in women. This finding is of major relevance as patients at high risk are those in whom a more precise prognosis is most important in terms of both resource allocation and treatment selection.

With regard to sex differences, and in accordance with previous findings (4–6, 24), in the MECKI score population, we observed a significantly lower LVEF in men compared to women (32.3% ± 9% vs. 36.3% ± 11%, respectively) and significant sex differences in NHYA class, etiology, and medical/device treatments. In our study population, 89.3% of women vs. 92.3% of men were treated with ACEi/ARBb/ARNI and only 24.72% and 11.5% of women vs. 35.54% and 14.96% of men were implanted with ICD and CRT, respectively. The underuse of CRT, which may be at least partially explained by the averaged higher LVEF in women than men among HFrEF patients, remains a matter of concern as left bundle branch block (BBS) is a more common finding in women compared to men (19), and it reinforces the theme of under-treatment in women with HF. Regardless, the overall risk in women was approximately half (9.79 vs. 4.11, respectively) of that in men. Indeed, the female sex has always represented a significant predictor of improved survival in patients with HFrEF, despite a comparatively low peakVO_2_ (11, 12). Nevertheless, after propensity score matching harmonization, the outcome advantage of the female sex vanishes, as shown in an early evaluation of the MECKI score dataset by Agostoni et al. (17). Similar findings were reported in the latest report of the American Heart Association, which showed an equal distribution of HF-related mortality between sexes, with a HF-related mortality of 46.6% across men and of 53.5% across women in 2019 (25).

For each variable included in the MECKI score since the original 2013 reports (17), prognostic significance was maintained across sexes using the updated model in the present study. In both sexes, the highest weight was for peakVO_2_ (Table 3). No discrepancy in weight was found for MDRD or VE/VCO_2_ between sexes, while a significant difference was estimated for the remaining variables (LVEF, Hb, Na^+^, and peakVO_2_) (Table 2). In particular, LVEF, Na^+^, and peakVO_2_ showed a higher impact in men, while Hb showed a greater weight in women. The higher impact of LVEF may be explained by a more frequent ischemic etiology of HF in men; of Na^+^ by an increased tendency to hyponatremia, possibly related to the greater use of diuretics and HF medications in men (26); and for peakVO_2_ by a lower value when it is reported as a percentage of the predicted value in men compared to women albeit the higher absolute value. The lower concentration of Hb typically observed in women along with the increased susceptibility to anemia and iron deficiency (27) may explain the greater weight of Hb in the female population. Moreover, the total mass of red blood cells is normally lower in women, meaning that same absolute loss of Hb in women compared to men represents a greater relative loss in women. Finally, due to differences in HF etiology, further studies are needed to detect if etiology has a role in the results reported.

The present study has some limitations. First, the MECKI score registry started as a retrospective study, but was developed in a prospective fashion. Second, patients were studied over a wide period of time, which therefore includes different treatments and follow-up strategies. This may raise doubts about the applicability of the present results to current HF patients; however, the AUC results were similar when we considered only patients recruited after 2010. Third, we analyzed the original MECKI score variables—peakVO_2_, VE/VCO_2_ slope, Na^+^, LVEF, Hb, and kidney function—by MDRD formula and did not evaluate whether other parameters have an independent prognostic role in female patients with HF. As a matter of fact, the only parameter of the several studied that adds prognostic power to the MECKI score was an undefinable anaerobic threshold (28).

In brief, we showed that sex-calculated MECKI, the S-MECKI, may constitute a further step in the prognostic assessment of patients with severe HFrEF and may contribute to refined patient selection for advanced treatments. The native MECKI score has already revealed a very good discriminative ability in HF higher than other common scores, such as HFSS, SHFM, and MAGGIC (28, 29), such that the most recent European guidelines on HF recommend the MECKI score (3). The S-MECKI score in the present study showed a slight but significant improvement in risk stratification, with a relevant increase in accuracy in identifying both male and female HF patients at the highest risk of events. Moreover, we showed that the weight of the MECKI score variables varies between men and women, and this must be considered in the overall patient assessments.

## Data Availability

The datasets presented in this study can be found in online repositories. The data can be found here: https://zenodo.org/records/12158305.

## Group members of MECKI Score Research Group

Other participants of the MECKI score group to be acknowledged are listed below:

• Armando Ferraretti: Ospedale di Foggia, Italy.
• Cristina Gussago: Cardiology, Department of Medical and Surgical Specialities, Radiological Sciences, and Public Health, Brescia, Brescia, Italy.
• Domenico Scrutinio: Division of Cardiology, Istituti Clinici Scientifici Maugeri, Institute of Cassano Murge, Bari, Italy.
• Donatella Bertipaglia: “S. Maugeri” Foundation, Tradate, Italy.
• Elisa Battaia, Michele Moretti: U.O. Cardiologia, S. Chiara Hospital, Trento, Italy.
• Francesca Pietrucci: Cardiologia Riabilitativa, Ospedali Riuniti, Ancona.
• Geza Halasz and Bruno Capelli: UOC Cardiologia, G da Saliceto Hospital, Piacenza, Italy.
• Giovanna Gallo and Emiliano Fiori: Department of Clinical and Molecular Medicine, Azienda Ospedaliera Sant’Andrea, “Sapienza” Università degli Studi di Roma, Roma, Italy.
• Giovanni Marchese: Cardiac Rehabilitation Unit, Istituti Clinici Scientifici Maugeri, Scientific Institute of Milan, Milan, Italy.
• Giuseppe Pacileo, Fabio Valente, Rossella Vastarella, and Rita Gravino: Cardiologia SUN, Ospedale Monaldi (Azienda dei Colli), Seconda Università di Napoli, Napoli.
• Matilda Shkoza: Ospedali Riuniti, Ancona, Italy.
• Nikita Baracchini, Teresa Capovilla, and Andrea Di Lenarda: Cardiovascular Department, Ospedali Riuniti and University of Trieste, Trieste, Italy.
• Alberto Maria Marra, Roberta D’Assante and Giulia Crisci: Federico II, Naples, Italy.
• Roberto Ricci and Luca Arcari: Cardiology Division, Santo Spirito Hospital, Roma, Italy.
• Sergio Caravita and Elena Viganò: Istituto Auxologico Italiano, Milan, Italy.
• Stefania Farina, Beatrice Pezzuto, Pietro Palermo, Mauro Contini, Paola Gugliandolo, Irene Mattavelli, and Michele Della Rocca: Centro Cardiologico Monzino, IRCCS, Milan, Italy.

## Supplementary material

Supplementary Figure 1

AUCs for each parameter of the MECKI score according to sex.
