# Supplementary figures and images for: A matter of sex—persistent predictive value of MECKI score prognostic power in men and women with heart failure and reduced ejection fraction: a multicenter study
Source: Front Cardiovasc Med. 2024 Jul 3;11:1390544. doi: 10.3389/fcvm.2024.1390544 (PMC11253175; doi:10.3389/fcvm.2024.1390544)

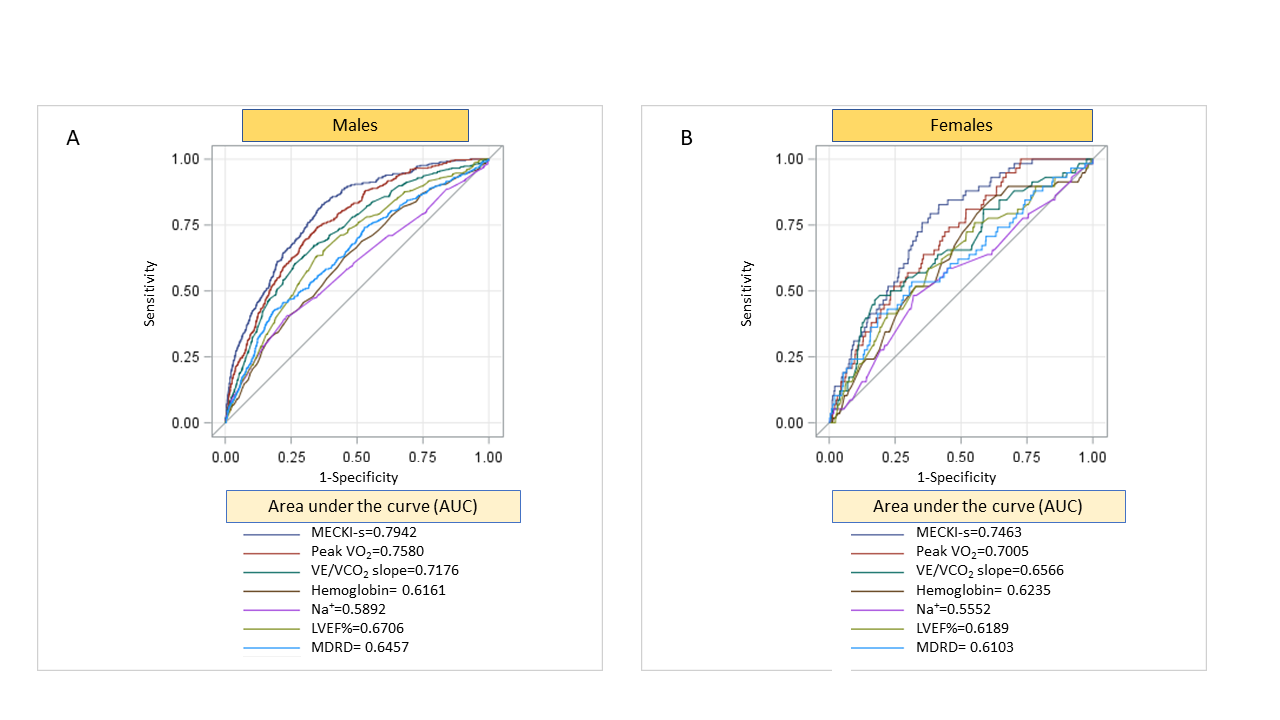

Supplement: Supplementary file 1 [file Image1.tif]
